# Reference values for estimated VO_2_max by two submaximal cycle tests: the Åstrand-test and the Ekblom-Bak test

**DOI:** 10.1007/s00421-023-05398-8

**Published:** 2024-01-22

**Authors:** Daniel Väisänen, Björn Ekblom, Peter Wallin, Gunnar Andersson, Elin Ekblom-Bak

**Affiliations:** 1 The Swedish School of Sport and Health Sciences, Stockholm, Sweden; 2 Department of Research, HPI Health Profile Institute, Danderyd, Stockholm, Sweden

**Keywords:** VO_2_max, Cardiorespiratory fitness, Reference values, Submaximal test

## Abstract

**Aims:**

Submaximal tests estimating VO_2_max have inherent biases; hence, using VO_2_max estimations from the same test is essential for reducing this bias. This study aimed to establish sex- and age-specific reference values for estimated VO_2_max using the Åstrand-test (Å-test) and the Ekblom-Bak test (EB-test). We also assessed the effects of age, exercise level, and BMI on VO_2_max estimations.

**Methods:**

We included men and women (20–69 years) from the Swedish working population participating in Health Profile Assessments between 2010 and 2020. Excluding those on heart rate-affecting medicines and smokers, n = 263,374 for the Å-test and n = 95,043 for the EB-test were included. VO_2_max reference values were based on percentiles 10, 25, 40, 60, 75, and 90 for both sexes across 5-year age groups.

**Results:**

Estimated absolute and relative VO_2_max were for men 3.11 L/min and 36.9 mL/min/kg using the Å-test, and 3.58 L/min and 42.4 mL/min/kg using the EB-test. For women, estimated absolute and relative VO_2_max were 2.48 L/min and 36.6 mL/min/kg using the Å-test, and 2.41 L/min and 35.5 mL/min/kg using the EB-test. Higher age (negative), higher exercise level (positive), and higher BMI (negative) were associated with estimated VO_2_max using both tests. However, explained variance by exercise on estimated VO_2_max was low, 10% for the Å-test and 8% for the EB-test, and moderate for BMI, 23% and 29%.

**Conclusion:**

We present reference values for estimated VO_2_max from two submaximal cycle tests. Age, exercise, and BMI influenced estimated VO_2_max. These references can be valuable in clinical evaluations using the same submaximal tests.

**Supplementary Information:**

The online version contains supplementary material available at 10.1007/s00421-023-05398-8.

## Introduction

Maximal oxygen uptake (VO_2_max) is a physiological indicator of an individual’s cardiorespiratory fitness and represents the maximum rate of the cardiovascular and respiratory systems in delivering oxygen to the working muscles. VO_2_max can be expressed in absolute (L O_2_/min) and relative (mL O_2_/min/kg) terms. Relative VO_2_max has been widely recognized as a measure for assessing health status, predicting cardiovascular disease risk, and evaluating athletic performance (Bassett and Howley 2000; Myers et al. 2002).

Two factors that can impact VO_2_max are exercise level and body mass index (BMI). Exercise level may directly impact VO_2_max, as regular physical activity of sufficient intensity have been shown to increase an individuals VO_2_max (Aadahl et al. 2007). Also, BMI may also have impact on VO_2_max level with a higher BMI often correlating with a lower VO_2_max (Mondal and Mishra 2017).

Traditionally, direct measurement of VO_2_max requires participants to undergo maximal exercise testing on a graded treadmill or cycle ergometer until voluntary exhaustion. While this approach provides accurate and precise results, it is often not feasible due to restrictions to laboratory conditions and expertise and health risks in mixed nonathlete populations.

To address these limitations, various methods have been developed to estimate VO_2_max using submaximal exercise tests (Noonan and Dean 2000). These tests often involve standardized practices where variables such as heart rate, power output, time, speed, or distance are measured. By extrapolating the submaximal data using predictive equations or nomograms, VO_2_max can be estimated.

Despite the availability of several norm predictions for estimating VO_2_max, translating these general norms into specific tests could introduce bias (Noonan and Dean 2000). Normative values derived from diverse populations may not accurately reflect the characteristics of the individuals being tested, introducing inherent error and potentially affecting the validity of the estimated VO_2_max values. Additionally, test-specific norms based on large, well-defined samples are needed to ensure accurate and reliable estimation of VO_2_max.

Two popular submaximal cycle ergometer tests are the Åstrand test (Å-test) (Astrand and Ryhming 1954; Astrand 1960) and the Ekblom-Bak test (EB-test) (Ekblom-Bak et al. 2014; Björkman et al. 2016). While the EB-test uses the change in heart rate response between two submaximal workloads, each for 4 min, the Å-test utilizes the heart rate response to cycling on a single submaximal workload for 6 min. These tests have gained popularity due to their simplicity, feasibility, and ability to estimate VO_2_max without requiring maximal exertion.

However, these tests may have some inherent biases against directly measured VO_2_max. Therefore, this study aims to provide test-, sex- and age-specific reference values for estimated VO_2_max using the Å-test and the EB-test, respectively, in a diverse population. A second aim was to study the influence of age, exercise level, and BMI.

## Method

Data was obtained from the HPI Health Profile Institute database, which includes Health Profile Assessments (HPAs) performed in the Swedish working population since the 1980-ies. An HPA includes a self-reported lifestyle questionnaire, anthropometric measurements, resting blood pressure evaluation, a submaximal cycle test, and concludes with a session with a health coach. HPAs are voluntarily accessible and free for employees in companies offering occupational health services. Historically, the Å-test, developed in the early 1960s, was the standard in HPAs. However, the EB-test, introduced in 2014, became an alternative in recent years.

For a contemporary study population mirroring current estimated VO_2_max levels, we included Å-tests and/or EB-tests from Swedish working individuals aged 20–69, conducted between 2010 and 2022. Out of a total sample of 481,815 men and women, a total of 370,880 had either performed an EB-test or an Å-test. Participants reporting intake of medicine that could affect heart rate response to physical activity were excluded. The resulting samples were for the Å-test n = 263,374 and for the EB test n = 95,043. As the output variables from the EB-test (sex, age, and heart rate response on the higher workload) can be used for calculation of estimated VO_2_max using the Å-test nomogram, n = 85,094 of the participants performing the EB-test also contributed with an estimated VO_2_max by the Å-test (Astrand and Ryhming 1954). See Supplement Figs. 1–3 for flowcharts.

### The Åstrand test

The Å-test is based on measuring steady-state heart rate during the last minute of six-minute submaximal cycling on constant work rate (pedal frequency 50 rpm), aiming to obtain a rating of perceived exertion (RPE) of≈13 on the Borg’s scale (Borg 1970). VO_2_max is then estimated from a nomogram using workload, steady state heart rate, and sex, and further age-corrected (Astrand and Ryhming 1954; Astrand 1960). In a previous validation study, the mean (95%CI) difference between measured and estimated VO_2_max by the Å-test was -0.07 L/min (− 0.21 to − 0.06), and the coefficient of variance was 18.1% for men and women combined. Sex-specific analyses showed that men were being underestimated, 0.41 L/min (− 0.61 to − 0.20), coefficient of variance 14.8%, and women overestimated, 0.13 L/min (− 0.02 to 0.28), coefficient of variance 17.2%.

### The Ekblom-Bak test

The EB-test uses the change in heart rate response between two four-minute submaximal workloads (pedal frequency 60 rpm), where cycling on a standard rate with a resistance of 0.5 kiloponds precedes a higher, individually chosen work rate to obtain an RPE of 13–14 on the Borg’s scale (Borg 1970). Mean heart rate values are calculated by measuring heart rate every fifteen seconds during the final minute of each workload. VO_2_max is estimated using the sex-specific prediction EB-test equations (Björkman et al. 2016). In a cross-validation study, the EB-test showed no significant difference on group level between measured and estimated absolute VO_2_max, mean (95% CI) of difference 0.02 (− 0.04 to 0.08) and coefficient of variance 9.4% for men and women combined (Bjorkman et al. 2016). Men experienced a small overestimation by the EB-test, 0.11 (0.02 to 0.20) and coefficient of variance 8.3%, and women a small underestimation, − 0.09 (− 0.16 to − 0.01) and coefficient of variance 10.0%.

### Other measurements

Body mass and height measurements were acquired using standard methods, with individuals wearing lightweight clothing. BMI was determined using the formula: weight in kilograms divided by square height in meters (kg/m^2^). Exercise level was self-reported as weekly exercise frequency to maintain or improve physical fitness, health, using the following options: ‘Never,’ ‘Sometimes,’ ‘1–2 times/week,’ ‘3–5 times/week,’ or ‘At least 6 times/week,’ with individuals specifying their exercise frequency to maintain or improve physical fitness, health, and well-being. Exclusion criteria were smoking habits, categorized as ‘At least 20 cig/day,’ ‘11–19 cig/day,’ ‘1–10 cig/day,’ ‘Occasionally,’ or ‘Never.’ Only those reporting ‘Never’ were included. Additionally, self-reported medication usage for hypertension or those affecting heart rate and high blood pressure diagnoses were recorded as ‘yes’ or ‘no’.

### Statistics

The variables underwent visual normality inspection, revealing an approximation to a normal distribution. Consequently, we report the mean and standard deviation (SD). For analyzing differences between men and women, we employed independent t-tests. Additionally, to assess the effect size of these differences, we calculated Cohen’s d. Reference categories for relative estimated VO_2_max, segmented by 5-year age groups, were defined using percentiles: 0–10 (Very low), 11–25 (Low), 26–40 (Somewhat low), 41–60 (Average), 61–75 (Somewhat high), 76–90 (High), and 91–100 (Very high). Density plots were employed to provide smoothed probability density estimates, comparing the age-related distributions of the Å-test and EB-test in 10-year age groups. To explore associations between estimated VO_2_max and exercise and BMI, overall trends and percent of variance explained (R^2^) were used. These R^2^ values were derived from crude and sex- and age-adjusted generalized additive models with integrated smoothness estimation, utilizing five knots for the VO_2_max-BMI relationship. Additionally, crude and sex- and age-adjusted ordinary least squares regression was applied for the exercise-VO_2_max relationship.

The sample was divided into 10-year age groups (20–29, 30–39, 40–49, 50–59, 60–69) to assess the association with age. After that, the percentual difference between mean estimated VO_2_max of the current and the previous 10-year age group was calculated according to the following equation; (mean_previous decade_ − mean_current decade_)/mean_previous decade_ × 100 per decade.

All data handling, figures, and statistical analyses were performed with R version 4.2.0 Vigorous Calisthenics and the package tidyverse, and the flowcharts were made with the package dtrackr.

## Results

Table 1 shows the study population’s characteristics. Estimated absolute and relative mean (SD) VO_2_max was 2.84 ± 0.76 L/min and 36.8 ± 10.0 mL/min/kg for the Å-test, and 3.10 ± 0.74 L/min and 39.5 ± 8.5 mL/min/kg for the EB-test. Men exhibited significantly higher absolute estimated VO_2_max compared to women for both the EB-test (3.58 L/min vs. 2.41 L/min, p < 0.001, Cohen’s d = 2.470) and the Å-test (3.11 L/min vs. 2.48 L/min, p < 0.001, Cohen’s d = 0.899), and also significantly higher relative VO_2_max according to the EB-test (42.4 mL/min/kg vs. 35.5 mL/min/kg, p < 0.001, Cohen’s d = 0.910). However, for the Å-test, while a statistically significant difference was observed (36.9 mL/min/kg for men vs. 36.6 mL/min/kg for women, p < 0.001), the effect size (Cohen’s d = 0.030) was negligible. Tables 2 and 3 present the sex- and age-specific reference categories.

**Table 1:** Characteristics of the study populations

	N		Weight (kg)		Height (cm)		BMI		No/irregular weekly exercise	
	Å-test	EB-test	Å-test	EB-test	Å-test	EB-test	Å-test	EB-test	Å-test	EB-test
**Men**										
20–24 years	6459	2221	81.7 ± 14.0	82.2 ± 15.3	180.9 ± 6.8	180.9 ± 6.9	24.9 ± 3.9	25.1 ± 4.2	54%	53%
25–29 years	14,057	5449	83.5 ± 14.1	84.5 ± 15.0	181.3 ± 6.7	181.4 ± 6.8	25.4 ± 3.9	25.6 ± 4.2	47%	50%
30–34 years	17,606	6702	84.6 ± 13.9	85.2 ± 14.3	181.1 ± 6.8	181.1 ± 6.8	25.8 ± 3.9	26.0 ± 4.0	39%	42%
35–39 years	20,289	6757	85.7 ± 13.9	85.9 ± 14.3	180.7 ± 6.7	180.8 ± 6.7	26.2 ± 3.9	26.3 ± 4.1	34%	38%
40–44 years	23,254	7676	86.8 ± 13.9	86.9 ± 14.0	180.6 ± 6.6	180.8 ± 6.6	26.6 ± 3.9	26.6 ± 4.0	34%	39%
45–49 years	23,302	8360	87.8 ± 13.7	87.7 ± 13.8	180.4 ± 6.6	180.5 ± 6.7	26.9 ± 3.8	26.9 ± 3.9	36%	41%
50–54 years	20,707	8535	87.9 ± 13.2	88.7 ± 13.8	180.1 ± 6.6	180.3 ± 6.6	27.1 ± 3.7	27.3 ± 3.9	37%	41%
55–59 years	15,046	6437	87.1 ± 12.6	88.3 ± 13.2	179.7 ± 6.5	179.9 ± 6.6	27.0 ± 3.5	27.3 ± 3.7	36%	39%
60–64 years	9033	3785	85.5 ± 11.8	86.8 ± 12.1	179.0 ± 6.4	179.3 ± 6.5	26.7 ± 3.3	27.0 ± 3.5	35%	37%
65–69 years	1136	491	83.9 ± 11.5	85.0 ± 11.5	178.7 ± 6.3	178.9 ± 6.6	26.3 ± 3.2	26.6 ± 3.4	37%	41%
**Women**										
20–24 years	3903	1083	66.1 ± 12.2	68.3 ± 13.6	167.3 ± 6.2	167.2 ± 6.2	23.6 ± 4.1	24.4 ± 4.7	58%	56%
25–29 years	9213	3461	66.8 ± 12.5	67.8 ± 12.9	167.6 ± 6.4	167.5 ± 6.4	23.8 ± 4.2	24.1 ± 4.3	48%	50%
30–34 years	11,780	4200	67.8 ± 12.9	69.2 ± 13.8	167.4 ± 6.3	167.4 ± 6.4	24.2 ± 4.5	24.7 ± 4.8	39%	43%
35–39 years	15,203	4830	68.7 ± 12.9	69.1 ± 13.4	167.2 ± 6.2	167.2 ± 6.2	24.6 ± 4.4	24.7 ± 4.6	36%	40%
40–44 years	18,178	5959	70.0 ± 13.0	70.5 ± 13.4	167.1 ± 6.1	167.3 ± 6.2	25.1 ± 4.5	25.2 ± 4.6	41%	44%
45–49 years	18,269	6255	70.9 ± 13.0	71.6 ± 13.3	167.0 ± 6.2	167.1 ± 6.3	25.4 ± 4.4	25.6 ± 4.5	45%	46%
50–54 years	15,856	5933	70.9 ± 12.4	71.7 ± 12.7	166.7 ± 6.1	167.1 ± 6.2	25.5 ± 4.3	25.7 ± 4.5	47%	49%
55–59 years	12,058	4282	70.0 ± 11.6	70.9 ± 12.5	166.1 ± 5.9	166.6 ± 5.9	25.4 ± 4.0	25.5 ± 4.3	44%	47%
60–64 years	7272	2369	69.0 ± 10.8	69.9 ± 11.5	165.6 ± 5.7	165.9 ± 5.9	25.2 ± 3.8	25.4 ± 4.1	41%	42%
65–69 years	753	258	68.1 ± 10.5	69.8 ± 11.0	165.3 ± 6.1	165.5 ± 6.3	24.9 ± 3.8	25.5 ± 4.0	45%	46%

**Table 2:** Age and sex-specific reference values (in mL/min/kg) for the Å-test

	Very low	Low	Somewhat low	Average	Somewhat high	High	Very high	N
Percentile	0–10	11–25	26–40	41–60	61–75	76–90	91–100	
	**Men** (n = 150,889)							
20–24 years	≤ 29.8	29.9–35.3	35.4–39.1	39.2–44.1	44.2–48.7	48.8–56.0	≥ 56.1	6 459
25–29 years	≤ 29.8	29.9–35.0	35.1–39.0	39.1–44.3	44.4–49.1	49.2–56.6	≥ 56.7	14 057
30–34 years	≤ 28.4	28.5–33.3	33.4–37.2	37.3–42.3	42.4–47.1	47.2–54.8	≥ 54.9	17 606
35–39 years	≤ 27.0	27.1–31.8	31.9–35.5	35.6–40.4	40.5–45.1	45.2–52.1	≥ 52.2	20 289
40–44 years	≤ 25.9	26.0–30.5	30.6–34.1	34.2–38.9	39.0–43.2	43.3–50.0	≥ 50.1	23 254
45–49 years	≤ 25.0	25.1–29.3	29.4–32.8	32.9–37.3	37.4–41.5	41.6–47.9	≥ 48.0	23 302
50–54 years	≤ 23.8	23.9–27.8	27.9–31.0	31.1–35.3	35.4–39.2	39.3–45.3	≥ 45.4	20 707
55–59 years	≤ 22.6	22.7–26.5	26.6–29.5	29.6–33.7	33.8–37.1	37.2–42.8	≥ 42.9	15 046
60–64 years	≤ 22.0	22.1–25.4	25.5–28.4	28.5–32.1	32.2–35.5	35.6–40.7	≥ 40.8	9 033
65–69 years	≤ 21.5	21.6–25.0	25.1–27.5	27.6–30.9	31.0–34.0	34.1–39.1	≥ 39.2	1 136
	**Women** (n = 112,485)							
20–24 years	≤ 30.3	30.4–35.3	35.4–39.2	39.3–44.4	44.5–49.2	49.3–56.9	≥ 57.0	3 903
25–29 years	≤ 30.2	30.3–35.5	35.6–39.3	39.4–44.9	45.0–50.0	50.1–57.9	≥ 58.0	9 238
30–34 years	≤ 28.8	28.9–33.8	33.9–37.7	37.8–43.1	43.2–48.2	48.3–55.6	≥ 55.7	11 777
35–39 years	≤ 27.0	27.1–31.9	32.0–35.7	35.8–40.7	40.8–45.3	45.4–52.6	≥ 52.7	15 202
40–44 years	≤ 25.6	25.7–30.2	30.3–34.0	34.1–38.8	38.9–43.2	43.3–50.0	≥ 50.1	18 178
45–49 years	≤ 24.3	24.4–28.9	29.0–32.4	32.5–37.1	37.2–41.3	41.4–48.0	≥ 48.1	18 266
50–54 years	≤ 23.1	23.2–27.4	27.5–30.6	30.7–35.1	35.2–39.1	39.2–45.2	≥ 45.3	15 849
55–59 years	≤ 22.1	22.2–25.8	25.9–28.8	28.9–32.9	33.0–36.6	36.7–42.0	≥ 42.1	12 050
60–64 years	≤ 21.2	21.3–24.6	24.7–27.4	27.5–31.1	31.2–34.5	34.6–40.0	≥ 40.1	7 269
65–69 years	≤ 20.3	20.4–23.7	23.8–26.9	27.0–30.7	30.8–33.3	33.4–38.7	≥ 38.8	753

**Table 3:** Sex- and age-specific reference values (in ml/min/kg) for the EB test

	Very low	Low	Somewhat low	Average	Somewhat high	High	Very high	N
Percentile	0–10	11–25	26–40	41–60	61–75	76–90	91–100	
	**Men **(n = 56,413)							
20–24 years	≤ 39.7	39.8–45.3	45.4–49.0	49.1–53.4	53.5–56.8	56.9–61.6	≥ 61.7	2 221
25–29 years	≤ 38.0	38.1–43.4	43.5–46.8	46.9–50.7	50.8–54.1	54.2–58.6	≥ 58.7	5 449
30–34 years	≤ 36.5	36.6–41.2	41.3–44.5	44.6–48.6	48.7–51.8	51.9–56.4	≥ 56.5	6 702
35–39 years	≤ 35.5	35.6–39.6	39.7–42.8	42.9–46.5	46.6–49.7	49.8–54.3	≥ 54.4	6 757
40–44 years	≤ 34.1	34.2–38.0	38.1–41.0	41.1–44.7	44.8–47.7	47.8–52.0	≥ 52.1	7 676
45–49 years	≤ 32.9	33.0–36.7	36.8–39.4	39.5–42.8	42.9–45.6	45.7–49.9	≥ 50.0	8 360
50–54 years	≤ 31.3	31.4–34.8	34.9–37.4	37.5–40.6	40.7–43.4	43.5–47.6	≥ 47.7	8 535
55–59 years	≤ 29.7	29.8–33.1	33.2–35.5	35.6–38.4	38.5–40.9	41.0–44.7	≥ 44.8	6 437
60–64 years	≤ 28.4	28.5–31.6	31.7–33.9	34.0–36.5	36.6–38.8	38.9–42.6	≥ 42.7	3 785
65–69 years	≤ 27.9	28.0–30.6	30.7–32.5	32.6–35.0	35.2–37.7	37.8–41.7	≥ 41.8	491
	**Women** (n = 38,612)							
20–24 years	≤ 30.6	30.7–35.3	35.4–38.2	38.3–41.7	41.8–44.7	44.8–48.8	≥ 48.9	1 082
25–29 years	≤ 30.2	30.3–35.1	35.2–38.3	38.4–41.8	41.9–44.6	44.7–48.7	≥ 48.8	3 476
30–34 years	≤ 29.0	29.1–33.4	33.5–36.3	36.4–39.9	40.0–42.9	43.0–47.0	≥ 47.1	4 199
35–39 years	≤ 28.4	28.5–32.8	32.9–35.5	35.6–39.0	39.1–41.7	41.8–46.1	≥ 46.2	4 829
40–44 years	≤ 27.2	27.3–31.3	31.4–34.2	34.3–37.7	37.8–40.6	40.7–44.9	≥ 45.0	5 958
45–49 years	≤ 26.0	26.1–29.8	29.9–32.5	32.6–36.1	36.2–39.0	39.1–42.9	≥ 43.0	6 255
50–54 years	≤ 25.2	25.3–28.8	28.9–31.4	31.5–34.6	34.7–37.3	37.4–41.2	≥ 41.3	5 929
55–59 years	≤ 24.3	24.4–27.6	27.7–30.1	30.2–33.1	33.2–35.7	35.8–39.4	≥ 39.5	4 278
60–64 years	≤ 23.5	23.6–27.0	27.1–29.2	29.3–31.8	31.9–34.0	34.1–37.1	≥ 37.2	2 366
65–69 years	≤ 22.9	23.0–26.1	26.2–28.5	28.6–31.8	31.9–33.6	33.7–36.6	≥ 36.7	258

**Fig. 1:**
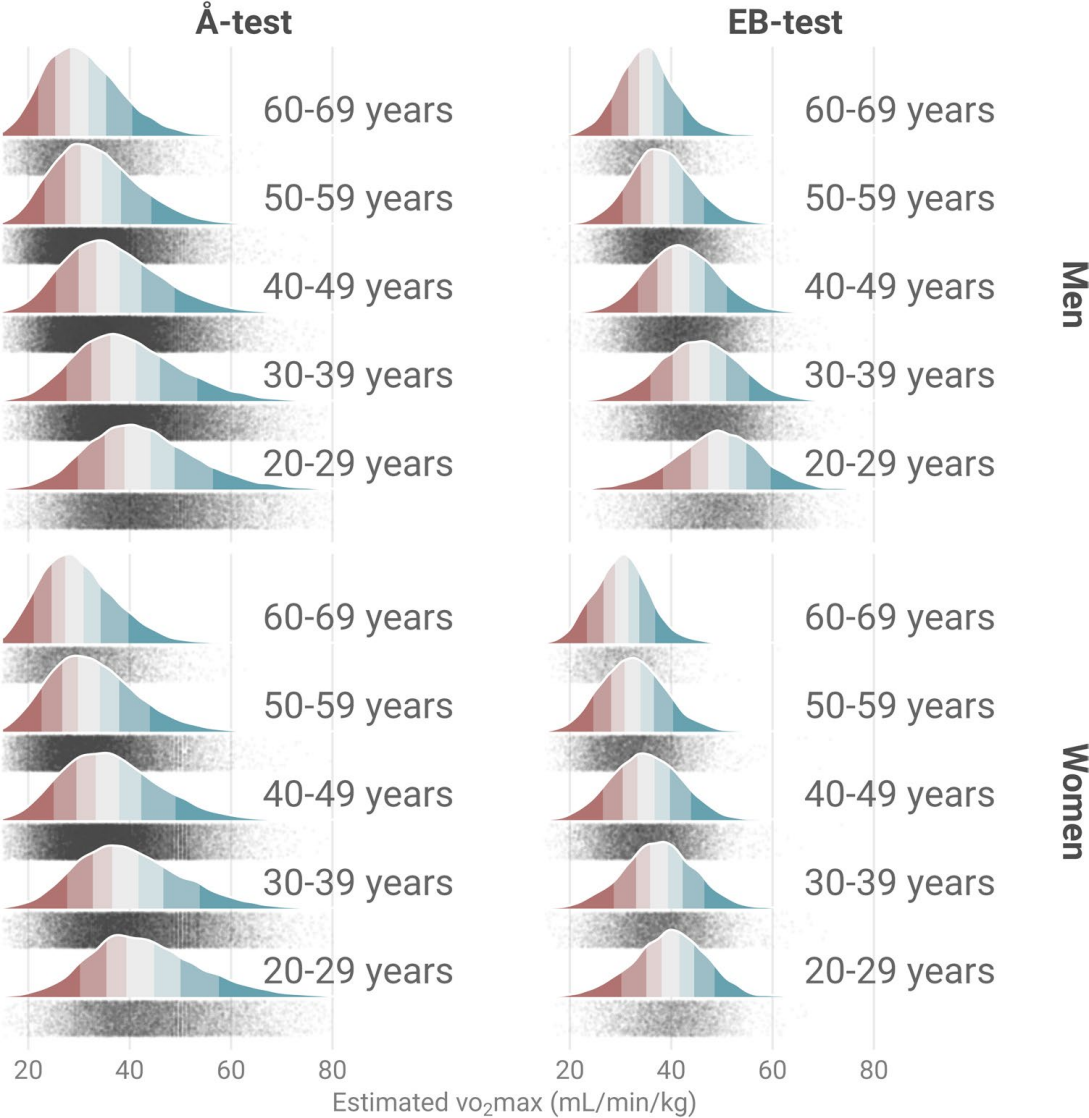
Density plot of estimated VO_2_max by the Å-test and the EB-test, respectively, in relation to 10-year age groups. Colors represent percentile groups in the order 0–10, 11–25, 26–40, 41–60, 61–75, 76–90 and 91–100

Relative VO_2_max was lower in higher 10-year age group (Fig. 1). The percentual difference of relative VO_2_max per 10-year age group compared to the previous (30–39 vs. 20–29 years, 40–49 vs 30–39 years, etc.) was for the the Å-test; men − 6.4%, − 7.8%, − 9.2%, − 7.6%, and women − 6.9%, − 9.3%, − 9.9% and − 9.0%. For the EB-test; men − 7.3%, − 7.8%, − 9.0%, and − 8.0%, and women − 5.2%, − 6.6%, − 7.5%, and − 6.9% for women.

**Fig. 2:**
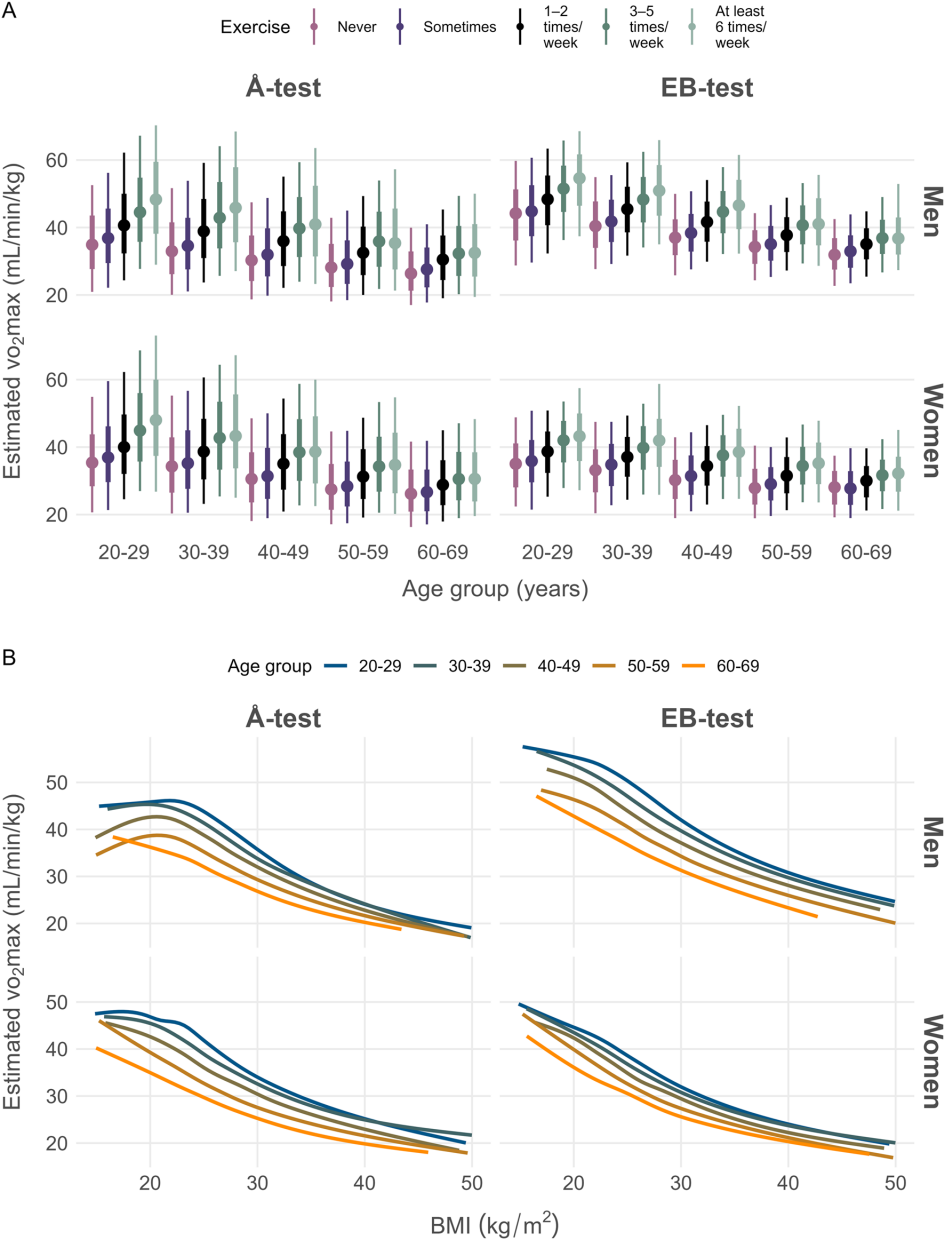
**A** The association between estimated VO2max and self-reported exercise level in relation to 10-year age groups. The middle point represents the median. The thicker line contains 66% of the study sample while the narow line contains 95%. **B** The association between estimated VO_2_max and BMI in relation to 10-year age groups

Estimated VO_2_max according to the Å-test as well as the EB-test were higher in participants reporting higher levels of exercise (p < 0.001 overall trend) (Fig. 2A). A model including sex, age, and self-reported exercise level explained 25% and 43% of the variance in estimated VO_2_max by the Å-test and the EB-test, respectively. The explained variance was 10% and 8% when including only self-reported exercise level.

Concerning BMI, estimated VO_2_max was lower with higher BMI, overall trend p = 0.001 for the Å-test and p < 0.001 for the EB-test (Fig. 2B). A model including sex, age, and BMI explained 34% and 67% of the variance in estimated VO_2_max by the Å-test and the EB-test, respectively, with an explained variance of 23% and 29% when only including BMI.

## Discussion

This study presents one of the most extensive reference samples for estimated VO_2_max values using two commonly used submaximal cycle ergometer tests. The main findings were that the EB-test generally indicated a higher mean estimated VO_2_max for men than women, while the Å-test showed comparable relative values between sexes. Estimated VO_2_max was lower with higher age for both tests. While individuals reporting more frequent weekly exercise had higher estimated VO_2_max using both tests, the explained variance was low. Also, higher BMI was associated with lower estimated VO_2_max, with moderate explained variance.

### Comparison of the two tests

The EB-test was developed with inspiration from the Å-test but aimed at reducing the quite large individual prediction error of the Å-test compared to direct measurements of VO_2_max (Ekblom-Bak et al. 2014; Bjorkman et al. 2016). Although both tests use a cycle ergometer and measure heart rate response to submaximal steady-state exercise, several differences between the tests may explain some of the variance between the reference values. Firstly, while the Å-test utilizes the heart rate response to cycling on one single submaximal workload for 6 min, the EB-test uses the change in (delta) heart rate response between two submaximal workloads, each for 4 min. This modification by the EB-test to use the change in heart rate response was one of the essential a priori principles that helped reduce the individual prediction error compared to the Å-test (Ekblom-Bak et al. 2014). While heart rate response to one single workload are influenced by both internal and external stimuli (nervousness, hot/cold temperature, stress, etc.), the delta change between two heart rate responses was shown to be more robust (Ekblom-Bak et al. 2014). Other diversities between the tests include, for example, the use of a hand-made nomogram with additional age-correction factors to estimate VO_2_max by the Å-test, while the EB-test equation was derived using computer-based regression modeling. Also, while the Å-test prediction model uses the assumption of linearity between heart rate and % of VO_2_max, the EB-test uses a logarithmic data-driven association between delta heart rate response and VO_2_max. Finally, the Å-test was developed based on data from a young, healthy population (men and women 18–30 years) (Astrand and Ryhming 1954), with age-correcting factors later developed to extend the use to older age groups (Astrand 1960). The EB-test was developed based on data from an already age-diverse population of men and women (20–86 years) (Björkman et al. 2016). Given the discrepancies between the two tests, tests that merely estimate rather than measure VO_2_max can introduce biases. Hence, references to be used for a test that estimates VO_2_max should be based on results from the same test.

### Comparison to directly measured VO_2_max

Loe et al. have presented one of the largest European reference materials of directly measured VO_2_max in 3816 men and women from the HUNT study, reporting mean absolute VO_2_max of 3.83 ± 0.72 L/min for men and 2.53 ± 0.49 L/min for women and relative VO_2_max of 45.4 ± 8.9 mL/min/kg for men and 37.0 ± 7.5 mL/min/kg for women (Loe et al. 2013b). This is somewhat higher than the estimated values in the present study. One main reason for differences in reference values derived from different populations is the population under study. Direct VO_2_max measurements demand maximal effort, potentially skewing recruitment towards younger and more fit individuals. This might account for the higher VO_2_max values in the HUNT study. Assessment mode also varies; VO_2_max is directly measured in maximal tests and estimated in submaximal ones. However, the laboratory equipment used when measuring maximal VO_2_max is not flawless. For example, the gas exchange analyzer used in the HUNT study (MetaMax) has been validated against the gold standard Douglas bag system with 8% higher VO_2_max values (Steene‐Johannessen et al. 2009). Compared to another Norwegian study with directly measured VO_2_max, the present study’s relative VO_2_max values were generally lower but closer to its average (Edvardsen et al. 2013). Yet, the EB-test and Å-test displayed higher VO_2_max values than American (Jackson et al. 1996; Talbot et al. 2000), Japanese (Sanada et al. 2007), and Brazilian populations (Herdy and Uhlendorf 2011). Based on a validation study (Bjorkman et al. 2016), these results indicate that the Å-test and the EB-test provide reliable average values for population-based research.

### Association of age, sex, exercise, and BMI to VO_2_max

A review including cross-sectional studies reports a 4%–12% lower relative VO_2_max per age-decade, with most studies displaying an approximately 10% lower relative VO_2_max per decade (Hawkins and Wiswell 2003; Letnes et al. 2023). The article further notes that a non-linear difference in VO_2_max occurs during the twenties and thirties in sedentary individuals. On the other hand, athletic individuals who reduce or stop their exercise habits experience a non-linear difference in cardiorespiratory fitness (Hawkins and Wiswell 2003). Authors of the HUNT study reported that the measured VO_2_max was specifically lower in the age group 40–49 years and older compared to younger age groups, similar to what was seen for the Å-test and the (Loe et al. 2013a). The Hunt study shows a relative decline per decade in VO_2_max (age group 20–29 years to 60–69 years) of 6.9% for men and 7.0% for women. Another extensive Norwegian study reported a per-decade lower measured VO_2_max of 8.3% in men and 7.2% in women. Notably, the VO_2_max values in the HUNT study were, on average, 9% higher than those in the other Norwegian study across all cohorts and both sexes (Edvardsen et al. 2013). This can be compared to the EB-test, where the difference in estimated VO_2_max by decade was relatively greater in men (− 7.1%) than women (− 5.9%). For the Å-test, the relation was opposite, − 6.9% for men and − 7.7% for women, which may be related to the fact that the Å-test test has been reported to underestimate VO_2_max in men and overestimate in women (Ekblom-Bak et al. 2014). These findings can be compared to other studies indicating that, in general, men tend to have higher VO_2_max levels than women (Sandvik et al. 1993; Hollenberg et al. 1998; Talbot et al. 2000; Fleg et al. 2005; Jackson et al. 2009; Wang et al. 2010; Herdy and Uhlendorf 2011). The higher VO_2_max in men than women is attributed to differences In muscle mass, hemoglobin levels, and cardiac stroke volume (Fletcher et al. 2013; Santisteban et al. 2022). Further, other studies have noted that the sex-based differences in cardiorespiratory fitness seem greater earlier in life and begin to narrow in elderly individuals (Hawkins and Wiswell 2003; Kaminsky et al. 2015).

Figure 2A illustrates that individuals who engage in higher levels of self-reported exercise tend to exhibit higher VO_2_max values, as observed for both the Å-test and the EB-test. This observation aligns with prior research findings that have consistently reported a positive correlation between exercise frequency and VO_2_max (Tager et al. 1998; Talbot et al. 2000; van Poppel et al. 2010; Loe et al. 2013a). However, the variance explained by exercise was relatively low in the present study (Å-test; R^2^ = 10%, EB-test; R^2^ = 8%), consistent with previous research findings (Tager et al. 1998; Talbot et al. 2000; van Poppel et al. 2010; Loe et al. 2013a). For instance, the HUNT study also demonstrated a limited overall fit between their Physical Activity Index and VO_2_max, yielding R^2^ values of 9% for men and 7% for women.

Both VO_2_max and BMI include body mass in their calculations, leading to an anticipated correlation. Our study confirmed this with a coefficient of determination showing R^2^ = 29% for the EB-test and R^2^ = 23% for the Å-test (Mondal and Mishra 2017). Additionally, our findings (Fig. 2B) for both the EB-test and Å-test indicate that higher BMI values are associated with lower relative estimated VO_2_max values, which has been reported by other research (Zeiher et al. 2019).

### Implications

The Å-test and the EB-test are practical and cost-effective means of estimating VO_2_max in large-scale studies. The tests can be easily implemented, allowing researchers to assess participants’ cardiorespiratory fitness easily. The estimated VO_2_max values can then be utilized to investigate associations between cardiorespiratory fitness levels and various health outcomes, such as chronic diseases, mortality, or cognitive function. Moreover, the reference values obtained from this study can serve as benchmarks for future research examining fitness trends in different populations or evaluating the impact of public health interventions on aerobic capacity.

The reference values established for estimated VO_2_max could also have implications for public health initiatives and fitness promotion. They aid in setting fitness goals, accounting for age and gender differences. Using estimated VO_2_max, health professionals can promote regular physical activity to enhance health and decrease chronic disease risk.

Further information of the Å-test may be found in the publications (Astrand 1960), while the EB-test has a public website: https://www.gih.se/ekblombaktest-english.

### Strengths and limitations

This study’s strengths include a vast and diverse sample covering both sexes and various ages from regions across Sweden, enabling detailed analysis of VO_2_max across age brackets. However, it’s not without limitations. The focus on the working population might limit its generalizability to non-working individuals. While using different test sites could present variability, standardized training provided by the HPI Health Profile Institute ensures consistent testing protocols.

## Conclusion

This study presents reference values for estimated VO_2_max using two commonly used submaximal cycle ergometer tests, the Å-test and the EB-test. While the EB-test indicated differences in estimated VO_2_max between men and women, estimated VO_2_max from the Å-test were similar between sexes. Age, exercise level, and BMI influenced the level of estimated VO_2_max. These test-specific reference values may be used in screenings and clinical practice to evaluate the estimated VO_2_max of an individual in relation to individuals of the same submaximal test, sex, and age.

### Supplementary Information

Below is the link to the electronic supplementary material.

Supplementary file1 (DOCX 564 KB)

## Data Availability

Data belongs to the HPI Health Profile Institute. Any data-inquiries are referred to them.

## Abbreviations

Å-test: Åstrand test
BMI: Body mass index
EB-test: Ekblom-Bak test
HPA: Health profile assessment
L O_2_/min: Liters of oxygen per minute
mL O_2_/min/kg: Milliliters of oxygen per minute per kilogram
RPE: Rating of perceived exertion
SD: Standard deviation
VO_2_max: Maximal oxygen uptake
