# Supplementary material for: Reference values for estimated VO2max by two submaximal cycle tests: the Åstrand-test and the Ekblom-Bak test
Source: Eur J Appl Physiol. 2024 Jan 22;124(6):1747–56. doi: 10.1007/s00421-023-05398-8 (PMC11129997; doi:10.1007/s00421-023-05398-8)
Supplement: Supplementary file 1 — Supplementary file1 (DOCX 564 KB) [file 421_2023_5398_MOESM1_ESM.docx]

**Supplementary Material**


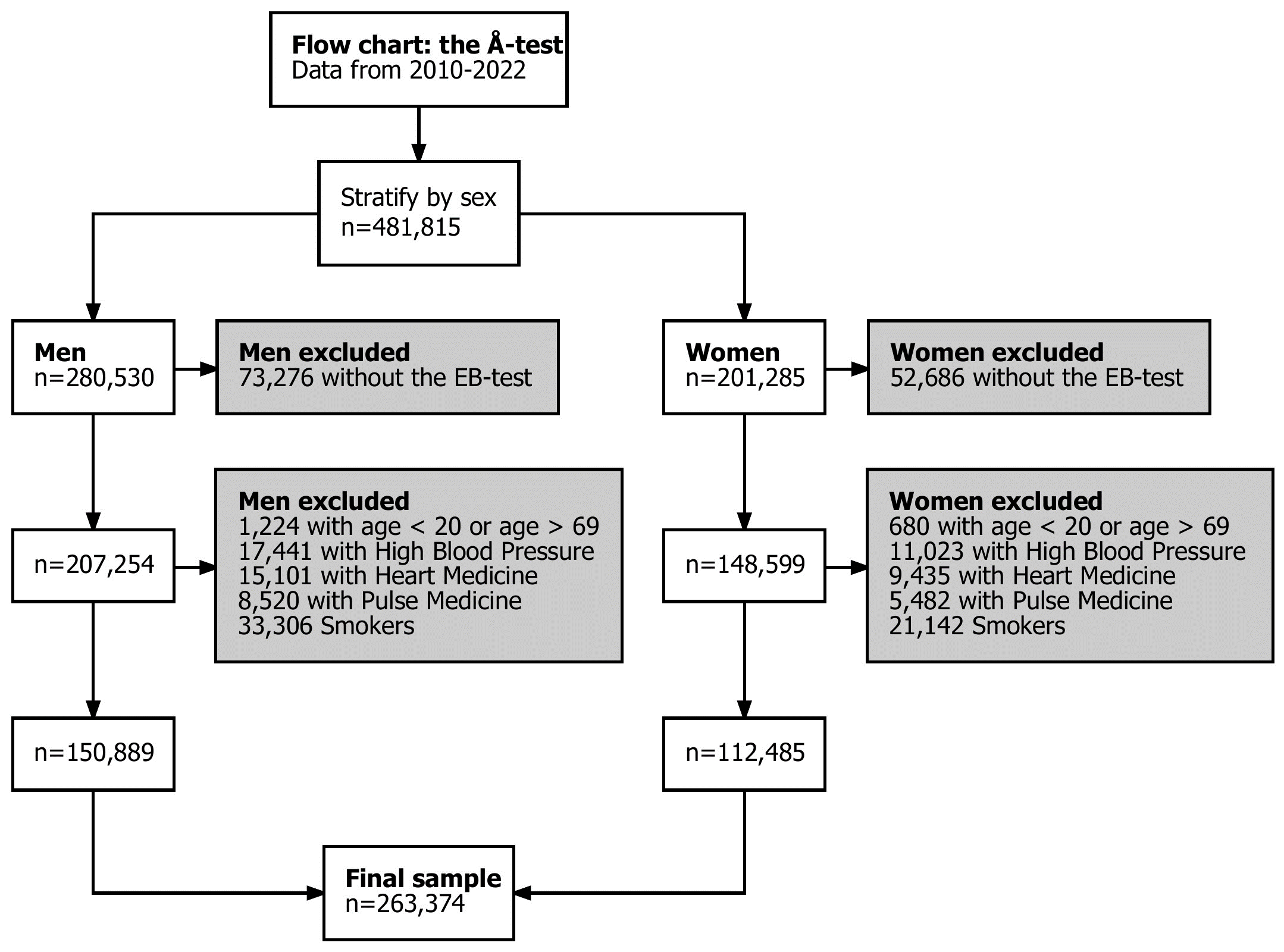


**Supplement Figure 1.** Flowchart of the Å-test data cleaning procedure. The number of excluded is not distinct for each criterion.


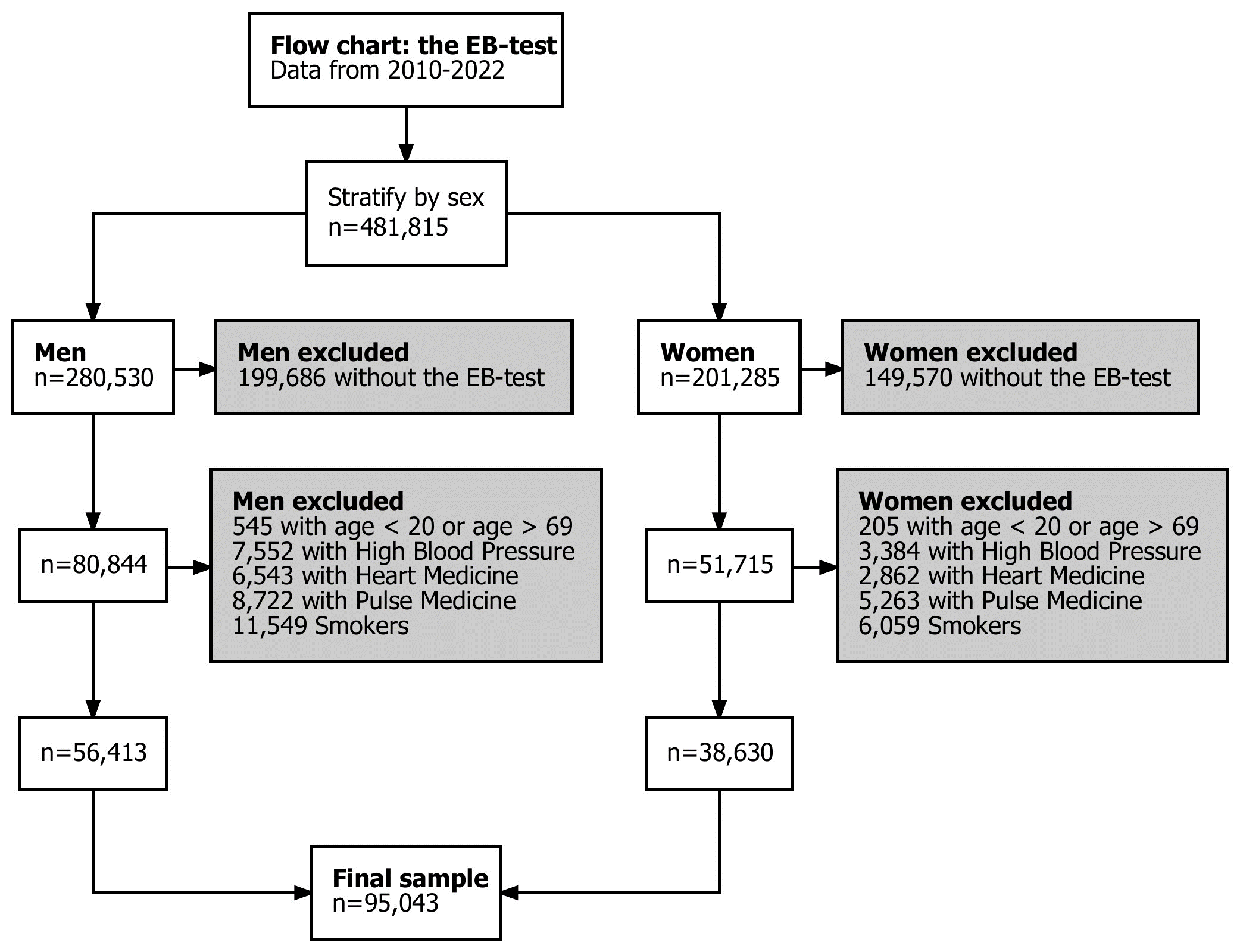


**Supplement Figure 2.** Flowchart of the EB-test data cleaning procedure. The number of excluded is not distinct for each criterion.


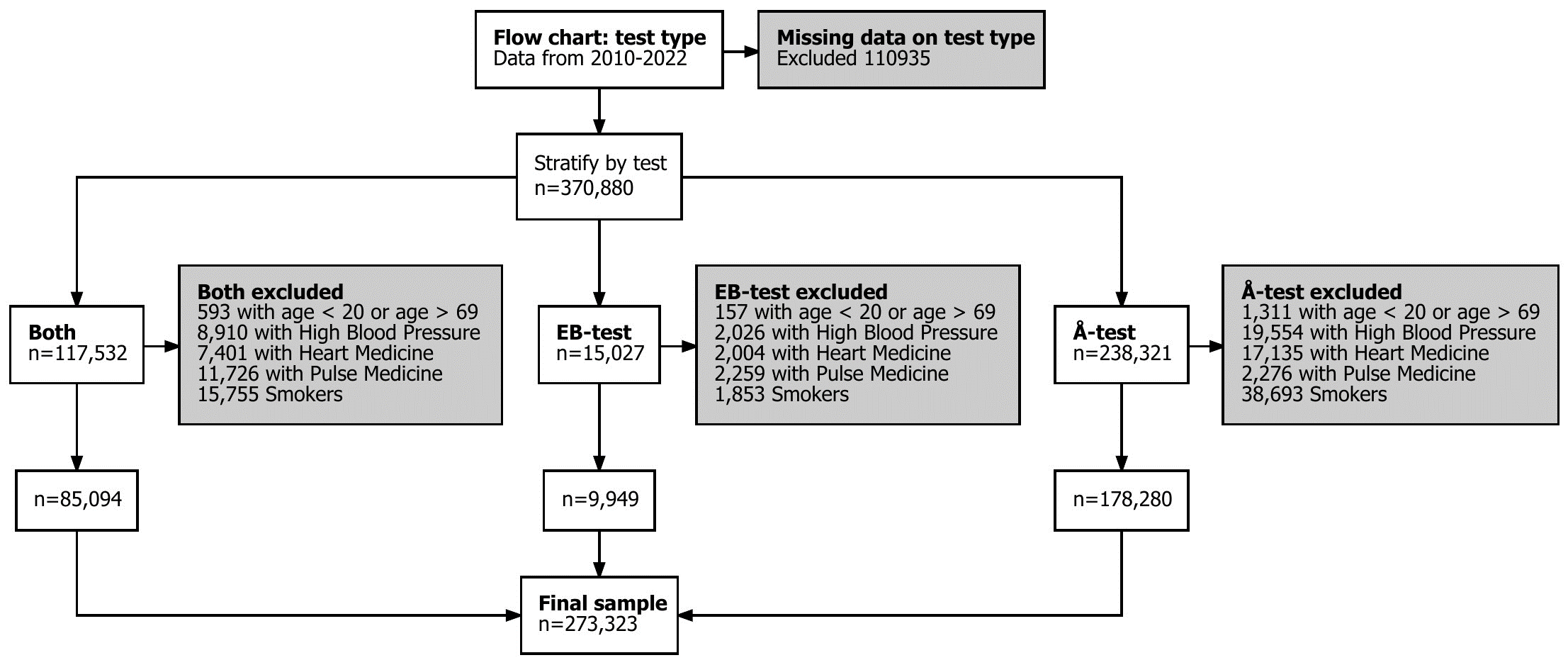


**Supplement Figure 3.** Flowchart of test type data cleaning procedure. The number of excluded is not distinct for each criterion.
